# Supplementary material for: Molecular imaging of humain hair with MeV-SIMS: A case study of cocaine detection and distribution in the hair of a cocaine user
Source: PLoS One. 2022 Mar 25;17(3):e0263338. doi: 10.1371/journal.pone.0263338 (PMC8956162; doi:10.1371/journal.pone.0263338)
Supplement: Attachment — Submitted filename: KN PLOS ONE Review Joe.docx [file pone.0263338.s001.docx]

The paper by Jeromel,L. et al looks at distribution of cocaine in human hair, using TOF-SIMS. The paper is well written and edited, and is recommended for publications. However the authors may clarify a few points listed below in the manuscript. These are suggestions only.

Somewhere in the introduction, the question regarding the standards such as cholesterol or the specific amino acids should be clarified. Why were these specific standards used, considering cholesterol is hydrophobic compared to the amino acids. Do these ions have any specific desorption rates compared to cocaine? Was free cocaine in solution form studied?

I am not sure how changes occur in “post mortem” state of the hair cells? Kindly clarify.

In the last figure the zones of high concentration (red) of cocaine at specific spots of the hair is due to especial location of the hair due to any structures of the hair? Why are the signals stronger from these specific areas? Kindly describe the figure in more details.
